# Fungal Keratitis Caused by *Humicola sardiniae*

**DOI:** 10.1007/s11046-024-00913-7

**Published:** 2024-12-23

**Authors:** Atsuhiko Fukuto, Kaori Mitoma, Keisuke Nakamura, Kayoko Tadera, Taiichiro Chikama, Takashi Yaguchi

**Affiliations:** 1 Department of Ophthalmology and Visual Sciences, Graduate School of Biomedical and Health Sciences, Hiroshima University, Hiroshima, 734-8551 Japan; 2 Section of Clinical Laboratory, Division of Clinical Support, Hiroshima University Hospital, Hiroshima, 734-8551 Japan; 3 Division of Laboratory Medicine, Hiroshima University Hospital, Hiroshima, 734-8551 Japan; 4 Medical Mycology Research Center, Chiba University, Chiba, 260-8673 Japan

**Fig. 1:**
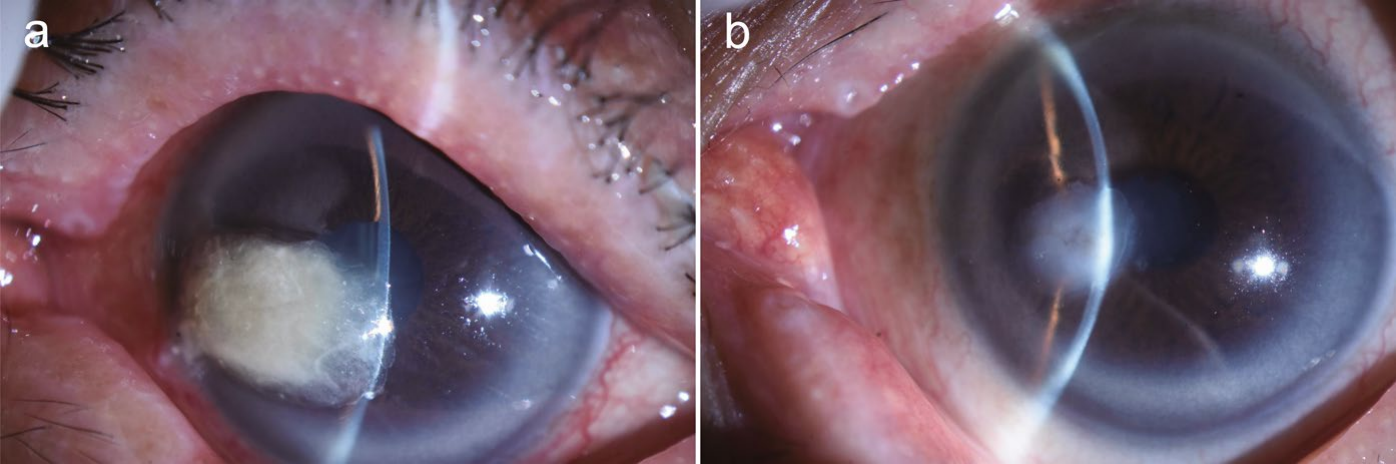
**a** Slit-lamp examination at the initial visit shows a 5-mm white mass adhered to the inferotemporal cornea, with a collapsed anterior chamber. **b** Two months after the initial visit, the corneal ulcer had re-epithelialized, and the anterior chamber had reformed

**Fig. 2:**
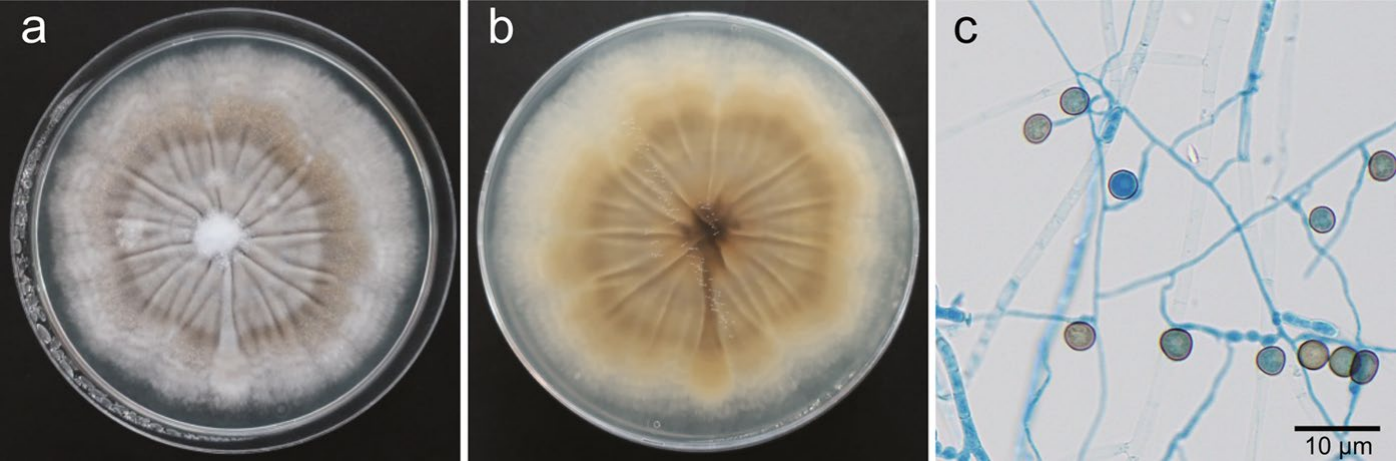
**a** A colony grown on potato dextrose agar at 30 °C for 14 days shows a grayish-white surface. **b** The reverse side of the colony is yellowish-brown. **c** Slide culture showing the formation of subglobose aleurioconidia (lactophenol cotton blue staining, × 400)

A 78-year-old man, who was receiving topical treatment for bilateral glaucoma, presented to a local clinic complaining of left eye pain and was prescribed 1.5% levofloxacin eye drops and 0.1% betamethasone phosphate eye drops. One month later, a white mass had adhered to the cornea, and natamycin eye drops were initiated. Filamentous fungi were detected in the culture of corneal scrapings. However, betamethasone phosphate eye drops were continued because their discontinuation led to a recurrence of redness and pain. Two months after the onset of symptoms, the patient was referred to a general hospital, where betamethasone phosphate eye drops were discontinued, and treatment was continued with 1.5% levofloxacin eye drops, 1% voriconazole eye drops, and 1% natamycin eye ointment. Although the lesion had reduced, the anterior chamber had collapsed, leading to a referral to our department 3 months after the initial onset. On a slit-lamp examination, a white mass was observed in the cornea of the left eye, and the anterior chamber had entirely collapsed (Fig. 1a). The white mass was removed and submitted for culture testing. Conservative treatment with forced eyelid closure was implemented because the corneal perforation was minor. Complete re-epithelialization was achieved 8 weeks after the initial presentation (Fig. 1b). The culture colony’s surface was grayish-white with a cottony texture (Fig. 2a), while the reverse side was yellowish-brown (Fig. 2b). Micromorphological examination showed hyphae with chlamydospores produced laterally or intercalary, which are thick-walled and brown, typical of the *Humicola* genus (Fig. 2c). The base sequences of the internal transcribed spacer region of rDNA and the β-tubulin gene showed 99.2% (497/501 bp) and 99.7% (386/387 bp) homology, respectively, with the *Humicola sardiniae* ex-type strain CBS 456.76 (GenBank accession numbers: MH444272 and MH444285). Therefore, we identified this isolate as *Humicola sardiniae*, and it was preserved as IFM 68935 at the Medical Mycology Research Center, Chiba University through the National Bio-Resource Project, Japan. Antifungal susceptibility testing was conducted using the CLSI M38-A2 standard. The minimum inhibitory concentrations obtained were as follows: micafungin, 2 µg/ml; caspofungin, 1 µg/ml; amphotericin B, 2 µg/ml; flucytosine, > 64 µg/ml; fluconazole, 8 µg/ml; itraconazole, 2 µg/ml; voriconazole, 2 µg/ml; and miconazole, 2 µg/ml.

*Humicola* belongs to the family *Chaetomiaceae* and is commonly found in soil and plant debris. Reports of human infection by *Humicola* species are sporadic. To the best of our knowledge, there have been no previous reports of *Humicola sardiniae* infection in humans, making this the first case reported globally. Information on the effectiveness of antifungal agents against *Humicola* species is limited. Hu et al. reported that itraconazole and voriconazole showed low minimum inhibitory concentrations against *Humicola pulvericola*. However, in the present antifungal susceptibility testing, the isolate resisted nearly all antifungal agents. Consequently, achieving a cure took four months from initiating antifungal treatment in this case.

